# HIV prevalence in recently incarcerated adult males in the Federal District, Brasilia, Brazil

**DOI:** 10.1590/0037-8682-0117-2019

**Published:** 2020-01-27

**Authors:** Rafael Jardim de Moura, Gustavo Adolfo Sierra Romero

**Affiliations:** 1 Universidade de Brasília, Núcleo de Medicina Tropical, Brasília, DF, Brasil.

**Keywords:** HIV, Prevalence, Rapid tests, Oral fluid, Prisoners

## Abstract

**INTRODUCTION:**

This study intends to describe a HIV intake screening strategy in recently incarcerated adults in Distrito Federal, Brasilia, Brazil.

**METHODS:**

We tested 455 recently incarcerated adults in Distrito Federal in 2016 using rapid tests (RT) applied to oral samples (OS).

**RESULTS:**

The estimated frequency of positive tests was 0.88% (95% confidence interval [CI] 0.34% to 2.24%).

**CONCLUSIONS:**

The present findings reveal the potential significance of detecting new HIV infection cases in a vulnerable population using point-of-care rapid diagnostic tests.

According to 2014 data, the Brazilian prison population was 607,731, and it has increased by 161% since 2000 - 10 times higher than the general population. If the same rate of incarceration is maintained in 2022, Brazil’s prison population will surpass the 1 million people mark. There are no official data regarding the number of people detained yearly, even briefly^1^. 

The majority of prisoners are young uneducated males, mostly between 18 and 29 years of age, arrested for robbery, burglary, drug dealing, and homicide^1^.

This population, mostly derived from disfavored communities, is usually affected by poor health and sanitary conditions even before incarceration. Unfavorable hygiene conditions, poorly ventilated prison cells, and overcrowding contribute to the worsening of their health status^2^. 

In a perverse manner, the justice system confines society members with higher risk of getting sick and who frequently do not seek health services. This provides an opportunity to improve public health efforts. For some detainees, this becomes the first contact with the health system^3^.

Regarding infectious diseases, intake screening should be an integral part of the prison system, because of transmission rates and high prevalence of diseases, such as HIV^2^ ^,^ ^3^. Under those conditions, instituting specific diagnostics and therapeutic measures might be beneficial, not only for the person being incarcerated, but also for the general community^4^.

Rapid tests are simple immunoassays that can be performed within 30 minutes. As they become more available, HIV diagnoses can be made away from labs, improving access. They have more than 99.5% sensitivity and 99% specificity for the detection of HIV^5^.

Considering that rapid HIV tests are approved in Brazil and recommended by the Ministry of Health for use in vulnerable populations, such as prison inmates^5^, the objective of this study was to describe the use of oral fluid rapid tests as an HIV intake screening strategy.

Candidates who were eligible to participate in this cross-sectional descriptive study were adult males recently arrested in the Federal District, Brasilia, Brazil, in 2016. Exclusion criteria were absence of cognitive capacity (influence of alcohol and/or drugs, major diseases, psychiatric conditions) for making an autonomous decision to sign the written consent. Potential candidates’ cognitive capacity was subjectively evaluated by the researcher. 

In the Federal District, individuals are taken to a centralized intake unit, "Carceragem do Departamento de Polícia Especializada (DPE)" after being arrested. An average of 40 people are admitted daily. The subjects were recruited at this unit, in a sequential manner, during weekdays, between July and August 2016.

Subjects were approached in a collective holding cell, where they were allocated immediately after arrival while waiting police identification procedures. They were informed that everyone would be tested but individuals were allowed to refuse (opt-out strategy). Those who refused were instructed to go to the back of the cell. A consent explanation ensued. Those willing to have the exam formed a queue for signing the written informed consent and receiving the oral swab. The sample collection instructions were provided collectively, but each individual collected his own sample. 

Subjects were informed of their results after 30 minutes, and those testing negative or indeterminate were instructed on how to proceed. 

The oral sample rapid test was “TR DPP HIV ½ - FO/BIO-MANGUINHOS,” lots 159RO047Z (good through 08/16) and 155RO045Z, (good through 09/16). The test was applied following the manufacturer’s instructions. 

This study followed ethical principles involving research with human beings in accordance with the National Health Council and was approved by the of the Department of Medicine of the University of Brasilia Ethics Committee (CAAE: 51813615.2.0000.5558**).**

We estimated a sample size of 455 individuals, considering a total population of arrested adult males of 15,000 in 2016, with 0.9% precision, 95% confidence level, and 1% expected HIV infection prevalence, using the formula n= [Z^2^p(1-p)]/e^2^ *,* where: z =1,96; p = 1%; e = 0,9%. Data were analyzed using the software Statistical Package for Social Sciences (SPSS version 22). 

Between 07/06/2016 and 08/18/2016, within 28 weekdays, HIV oral fluid rapid tests were offered to 618 recently incarcerated adult men, from which 455 consented (73.6%). No subjects were excluded. On average, 12.7 people were tested daily, with a minimum of nine and maximum of 25. 

Of the 455 individuals tested, 34.5% (n=157) remained only 1 day after being arrested and were released after presentation before a judge on the subsequent day of the arrest. Within 30 days, 59.6% (n=271) remained imprisoned, and 41.4% had been released (Figure 1).

**FIGURE 1:**
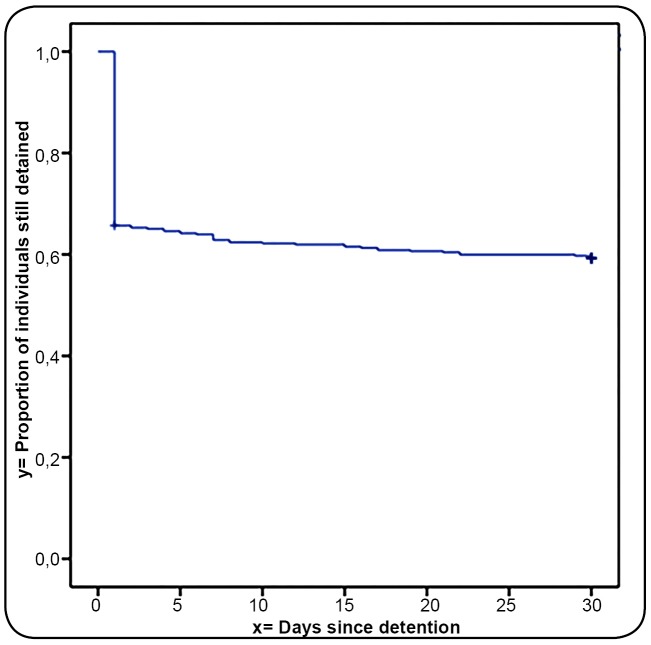
Kaplan-Meier survival curve with the proportion of individuals still detained within the first 30 days after arrest in the Federal District, Brasilia, Brazil between July and August 2016.

Age data were made available by the criminal authority for only 366 subjects (80.4%) who were not released by the judge or were previously convicted (Table 1). Mean age was 27.26 years (SD=9.02 years; minimum=18 and maximum=63 years). There were previous arrests records for 37.13% (n=169) of subjects. On average, 674.59 days had gone by since the previous release (SD=850 days; minimum 0 days and maximum 5,643 days).

**TABLE 1:** Demographic data of the 366 subjects who were not released by the judge on the day after arrest or who were previously convicted.

Variables	n (%)
Age [mean ± SD]	27.26 ± 9.02
Previously arrested	169 (37.13)
Days since the previous release [mean ± SD]	674.59 ± 850

**SD:** standard deviation.

Among the 455 subjects, four tested positive for HIV corresponding to an estimated frequency of 0.88% (95% CI 0.34% to 2.24%). Three were new cases. All cases that tested positive were still detained one month after incarceration, were later confirmed as HIV cases, and initiated treatment while detained.

As this was the first Brazilian study in a recently arrested population, there are no similar data for comparison. Considering other surveys among prisoners, a recent study in the Brazilian State Mato Grosso do Sul found an HIV prevalence of 1.54%^6^. 

Regarding the Brazilian general population, the estimated HIV prevalence was 0.4% in 2014^7^. Although the point frequency of the present study is higher, it is not possible to conclude that it is greater than the rate among the general population because of the precision of our estimate. 

There was a considerable testing rate (73.3%) even though the opt-out strategy was adopted. Compared to the opt-in strategy, in which the test is performed only after directly asking the person if he or she wants to be tested, in the opt-out method the examiner declares that the test will be given, providing the option to decline to participate. The opt-out method often yields a higher test rate^8^.

The choice of rapid test proved to be reasonable considering approximately 30% of the subjects were released the next day, which would have caused difficulty in delivering the results, considering the test options currently available^9^. 

There is controversy regarding whether the oral fluid rapid test sensitivity and specificity are similar to tests using finger or venous blood samples. A meta-analysis revealed as much as a 2% lower sensitivity in the oral sample tests^10^.

In regard to the immunoassays (ELISA) in venous samples, the new fourth generation tests, which also include antigen direct testing, have higher sensitivity for acute infections, which is of special interest for higher risk populations^11^. 

Rapid tests using finger blood samples and the conventional immunoassays still allow for multiple testing of other bloodborne diseases, such as syphilis and hepatitis B and C, and are also important in the imprisoned population^12^. 

There are reports of North American emergency services where venous sample ELISA results were available within three hours^13^. New immunoassay automated platform technologies deliver results in less than one hour^14^, which allows for testing more subjects simultaneously than would be feasible with individual rapid tests^14^.

Thus, considering the transitory nature of this population, the chosen opt-out strategy with the oral fluid rapid tests and dissociated individual pre-counseling, can be considered appropriate. Challenges remain regarding resources and more appropriate technologies.

Concerns about free consent and potential interference of a coercive environment^15^ remain challenging, but the testing rate of 73.3% reflects substantial refusal. The present study did not seek the reasons for refusal. A qualitative study could clarify such issues. Some of the subjects spontaneously reported that they had already been tested in recent detentions.

Potential discrimination of those testing positive is also a concern^15^, as there is a possibility of poor treatment outcomes and decreased adherence during detention and after release. In the author’s personal experience working as a clinician in the prison system, adherence and viral suppression are substantial. 

Detainees need access to screening and treatment of infectious diseases, such as HIV, upon entering the prison system. This is important not only for their own benefit but also for the community to which they will eventually return.

Research in the prison environment is always challenging. These challenges may include bureaucracy and the necessary cooperation of several stakeholders, including justice, security, and health personnel. Logistic issues include movement of the researcher within the facilities, lack of appropriate rooms for interviews, and unavailability of the prisoner due to justice appointments, meal time, cell transfers, courtyard time, lockdowns, and other security procedures.

Therefore, despite the need for additional studies on HIV prevalence, screening, prevention, and treatment in imprisoned populations, the present evidence demonstrates the usefulness of rapid tests using oral fluid as an accessible alternative for approaching this high-risk population.
